# *AIB1 *gene amplification and the instability of polyQ encoding sequence in breast cancer cell lines

**DOI:** 10.1186/1471-2407-6-111

**Published:** 2006-05-02

**Authors:** Lee-Jun C Wong, Pu Dai, Jyh-Feng Lu, Mary Ann Lou, Robert Clarke, Viktor Nazarov

**Affiliations:** 1 Department of Molecular and Human Genetics, Baylor College of Medicine, Houston, Texas 77030, USA; 2 Department of Otolaryngology, Head Neck Surgery, Chinese PLA General Hospital, Beijing 100853, China; 3 Fu Jen Catholic University, School of Medicine, Taipei, Taiwan; 4 Department of Surgery, Cardinal Tien Hospital, Hsintien Taipei Hsien, Taiwan; 5 Department of Oncology, Georgetown University Medical Center, Washington, DC 20007, USA

## Abstract

**Background:**

The poly Q polymorphism in *AIB1 *(amplified in breast cancer) gene is usually assessed by fragment length analysis which does not reveal the actual sequence variation. The purpose of this study is to investigate the sequence variation of poly Q encoding region in breast cancer cell lines at single molecule level, and to determine if the sequence variation is related to *AIB1 *gene amplification.

**Methods:**

The polymorphic poly Q encoding region of *AIB1 *gene was investigated at the single molecule level by PCR cloning/sequencing. The amplification of *AIB1 *gene in various breast cancer cell lines were studied by real-time quantitative PCR.

**Results:**

Significant amplifications (5–23 folds) of *AIB1 *gene were found in 2 out of 9 (22%) ER positive cell lines (in BT-474 and MCF-7 but not in BT-20, ZR-75-1, T47D, BT483, MDA-MB-361, MDA-MB-468 and MDA-MB-330). The *AIB1 *gene was not amplified in any of the ER negative cell lines. Different passages of MCF-7 cell lines and their derivatives maintained the feature of *AIB1 *amplification. When the cells were selected for hormone independence (LCC1) and resistance to 4-hydroxy tamoxifen (4-OH TAM) (LCC2 and R27), ICI 182,780 (LCC9) or 4-OH TAM, KEO and LY 117018 (LY-2), *AIB1 *copy number decreased but still remained highly amplified. Sequencing analysis of poly Q encoding region of *AIB1 *gene did not reveal specific patterns that could be correlated with *AIB1 *gene amplification. However, about 72% of the breast cancer cell lines had at least one under represented (<20%) extra poly Q encoding sequence patterns that were derived from the original allele, presumably due to somatic instability. Although all MCF-7 cells and their variants had the same predominant poly Q encoding sequence pattern of (CAG)_3_CAA(CAG)_9_(CAACAG)_3_(CAACAGCAG)_2_CAA of the original cell line, a number of altered poly Q encoding sequences were found in the derivatives of MCF-7 cell lines.

**Conclusion:**

These data suggest that poly Q encoding region of *AIB1 *gene is somatic unstable in breast cancer cell lines. The instability and the sequence characteristics, however, do not appear to be associated with the level of the gene amplification.

## Background

While predisposition to breast cancer is largely due to mutations in high penetrance tumor suppressor genes such as *BRCA1 *and *BRCA*2, progression of cancer is the result of accumulation of genetic alterations. These alterations include gene amplifications, microsatellite instabilities, loss of heterozygosity, and mutations in genes that play important roles in signal transduction or transcription activation pathways leading to tumorigenesis. Gene amplification in breast cancer was found in several chromosomal locations [1-4]. Among them, ErbB2 (or HER-2/neu) amplification strongly correlates with steroid receptor negative tumors [5,6], and amplification of *AIB1*(amplified in breast cancer 1) gene is prevalent in estrogen receptor (ER) positive tumors [7,8]. The *AIB1 *gene is a member of the SRC-1 (steroid receptor coactivator) family and is also known as RAC3, TRAM-1 or ACTR [7,9,10]. It is located at chromosome 20q12 region and encodes a protein of 1420 amino acids containing bHLH-PAS dimerization domain, a hormone receptor interaction domain, a CBP interaction domain, and histone acetyltransferase domain [11]. The amplifications and overexpression of *AIB1 *gene were found to be a common phenomenon in breast cancer cell lines and primary breast cancer tissues [12-15]. Since *AIB1 *bridges between nuclear receptors and other coactivators or the transcriptional machinery, its amplification and overexpression may play crucial roles in the development of breast cancer and may potentially have influence on the hormonal prevention and treatment for breast cancer.

Toward the C-terminus of AIB1, there is a stretch of polyglutamine residues that are encoded by polymorphic CAG repeats. The expansion of CAG repeats in poly Q containing proteins underlies a number of neurodegenerative diseases [16,17]. Large expansion of triplet repeats in *AIB1 *gene does not occur, presumably due to the frequent interruption by CAA [18]. However somatic instability by nucleotide substitution such as small insertion or deletion does occur [18]. In androgen receptor (AR), the length of the CAG repeats inversely correlates with its transcriptional activity [19,20]. Meanwhile a shorter CAG repeat in AR is associated with a higher risk of an aggressive prostate cancer phenotype characterized by extraprostatic extension, distant metastases, or poor histological grade [21]. In the case of AIB1, it is not clear if the polymorphic length of poly Q affects the transactivation activity of AIB1. AIB1 interacts with ER in a ligand-dependent manner [7]. It also interacts with non-steroid nuclear receptors and transcription co-integrators such as thyroid and retinoid receptors and CBP-dependent transcription complexes [22,23]. Thus, amplification of *AIB1 *gene impacts on both estrogen dependent and estrogen independent mechanisms leading to tumorigenesis [24-26]. Although antiestrogens are the most common type of endocrine therapy in breast cancer treatment, acquired resistance can be a major problem in clinical management of initially responsive breast cancer patients.

Understanding of the quantitative and qualitative changes of *AIB1 *gene in estrogen-independent and antiestrogen resistant breast cancer cell lines may help in the selection of steroid or non-steroid antiestrogen therapies. Evaluation of *AIB1 *gene amplification in previous reports is performed by FISH or Southern blot analysis [2,4,7]. In this report, we use the real-time quantitative PCR (Q-PCR) technique to assess the amplification of *AIB1 *gene in various breast cancer cell lines and primary breast tumors. We also analyze the sequence characteristics and instability of the polymorphic poly Q encoding region at the single molecule level by cloning and sequencing of the DNA region containing CAG repeats.

## Methods

### Samples and DNA preparation

Primary breast tumor specimens with matching normal breast tissue samples were obtained from Fu Jen Catholic University, and Cardinal Tien Hospital, Taiwan, after surgical removal of the tumor according to the IRB approved protocol. The ER positive breast cancer cell lines were obtained from Georgetown University Lombardi Comprehensive Cancer Center Tissue Culture shared resource and American Type Cell Culture. A total of 25 cancer and 4 non-cancer breast tissue cell lines were studied. MCF-7 variants include different passages, MCF-7 p19, MCF-7 p72 and MCF-7 derivatives: LCC1 (selected for growth *in vitro *without estrogens) [27], LCC2 (selected from LCC1 by treatment with non-steroid antiestrogen 4-OH TAM) [28], LCC9 (selected from LCC1 by treatment with steroid antiestrogen ICI 182,780) [29], LY-2 (resistant to 4-OH TAM, KEO and LY 117018) and R27 (resistant to 4-OH TAM). AK-47 is derived from parental ER positive cell line ZR-75-1 with the loss of expression of ER. LCC6 is a more aggressive form of MDA-MB-435 [30]. A1N4 is a normal breast cell line that is ER negative. DNA from tissues, blood and cell cultures was extracted by salting out method [31].

### Preparation of standard DNA for quantitative PCR

A region of 439 bp from exon 5 of *AIB1 *gene was amplified with the forward primer; 5'-CAAGCGATCAAATGAGGGTAG-3' and the reverse primer; 5'-CATTGTTTCATATCTCTGGCG-3'. A fragment of 85 bp from 3' untranslated region of *β*_2_*-microglobulin *gene (*β*_2_*-M*) was amplified with the forward primer; 5'-TGCTGTCTCCATGTTTGATGTATCT-3' and the reverse primer; 5'-TCTCTGCTCCCCACCTCTAAGT-3' [32-34]. These PCR products were cloned into the pCR 2.1-TOPO vector (Invitrogen). The plasmid DNA was isolated and quantified using the DU640 Spectrophotometer (Beckman, Fullerton, CA, USA). The copy numbers were calculated from absorbance at 260 nm and based on the molecular weight of the resulting plasmid. The plasmid DNAs were serially diluted over four logs to establish the standard curve giving a range from 400,000 to 40 copies/μl. In additional set of experiments the standard curve was constructed using genomic DNA prepared as a pool of equal amounts of blood DNA from 7 control individuals with normal *AIB1 *copy number. 'Normal' genomic DNA (100 ng/μl) was diluted in water over four logs. Since 1 ng of genomic DNA contains approximately 330 copies of a single copy gene, five standards used range from 33000 to 3.3 copies/μl.

### Real-time quantitative PCR (RT Q-PCR)

In real-time Q-PCR analysis, the primers used were 5'-GAGTTTCCTGGACAAATGAG-3' (forward) and 5'-CATTGTTTCATATCTCTGGCG-3' (reverse) for *AIB1 *gene (Exon 5), and the same primers as used for standard DNA preparation for *β*_2_*-M *gene, yielding 134 bp and 85 bp PCR products, respectively. The TaqMan probes were FAM-5' GCCGTATGTTGATGAAAACACCACA 3'-TAMRA and VIC-5' TTGCTCCACAGGTAGCTCTAGGAGG 3'-TAMRA, for *AIB1 *and *β*_2_*-M *gene respectively, each labeled with FAM or VIC (reporter dye) at the 5' end and TAMRA (quencher dye) at the 3' end. Each 10 μl real time Q-PCR reaction mixture contained 1 × TaqMan Universal PCR Master Mix (Applied Biosystems, Foster City, CA), 10 ng of genomic DNA, 0.3 μM of each primer, and 0.1 μM probe. The actin gene was also used as a reference. However, since the actin gene has multiple homologous copies, the data presented here were referenced to *β*_2_*-M *gene. The amplification was carried out according to the conditions suggested by the manufacturer (initial denaturation at 95°C for 10 min and 40 cycles of 95°C for 15 s and 60°C for 1 min) using an ABI Prism 7700 Sequence Detection System (Applied Biosystems, Foster City, CA). Each measurement was performed in triplicate and the threshold cycle numbers (C_T_) were measured. The copy number was generated from the C_T _value and standard curve according to previously described procedures [32-34].

### Cloning and sequencing

The poly Q containing fragment was amplified by the forward primer F: 5' GTCTTATACCTGGTGTATTG 3' and the reverse primer R: 5' CTGGGGGAAGCAGTCACATTAG 3', yielding a PCR product of 314 bp. The high fidelity amplification was carried out in a 30 μl reaction mixture containing 10 ng of genomic DNA, 0.2 μM of each primer, 1 × HF 2 PCR buffer, dNTPs, and Advantage-HF 2 polymerase according to the manufacturer's recommendation (Clontech Laboratories, Palo Alto, CA). After 1 min of initial denaturation at 94°C, the DNA was amplified by 30 cycles of 45 s at 95°C, 45 s at 55°C and 45 s at 72°C, followed by a final extension at 72°C for 5 min. The PCR products were purified and cloned into pCR2.1-TOPO (Invitrogen) vector according to the manufacturer's protocol. At least 8 clones from each sample were picked for sequencing using BigDye sequencing kit and analyzed on an ABI 377 DNA Sequencer (Applied Biosystems, Foster City, CA). Two primers, F and F2 (5' AGCAGGGTTTTCTTAATGCTC 3') were used for sequencing and loading of reactions onto alternate lanes for easy tracking. The sequence results were analyzed using sequence analysis software version 3.4.

## Results

### Amplification of AIB1 gene

Real-time Q-PCR analysis allows the measurement of actual copy number of *AIB1 *gene using a single copy *β*_2_*-microglobulin *gene as a reference. From the threshold cycle number and the standard curve, the ratio of the copy number of *AIB1 *gene to that of *β*_2_*-M *gene can be calculated. This ratio can be used as a measure of the amplification of the *AIB1 *gene. The average copy number ratio of the *AIB1*/*β*_2_*-M *in the blood samples from 48 age matched control individuals is determined to be 1.16 ± 0.38. An *AIB1*/*β*_2_*-M *ratio above 2 SD of the mean (1.16 + 2 × 0.38 = 1.92) is defined as truly amplified. In addition, all measurements were repeated using a pool of normal genomic DNAs for standard curve construction. The results obtained using both methods were practically identical.

We first evaluate the amplification of *AIB1 *gene in 26 primary breast tumors (13 ER positive and 13 ER negative) and corresponding surrounding normal breast tissue samples. *AIB1 *gene was found to be amplified in 1 ER positive tumor sample that constitutes 3.8% of total or 7.6% of ER positive tumors. This result is consistent with previous report [3,7,13].

As shown in Table 1, 9 out of 29 cell lines had elevated *AIB1 *at 2SD above the mean. All of them were ER positive. *AIB1 *gene was not amplified in 7 ER positive cell lines: BT20, ZR-75-1, T47D, BT483, MDA-MB-361, MDA-MB-468 and MDA-MB-330. None of the 13 ER negative cell lines showed significant *AIB1 *amplification. Some cell lines are derivatives of others. For example, AK-47 is derived from ER positive ZR-75-1 cell line. In AK-47 cells, loss of ER expression did not have any effect on *AIB1 *copy number. The amplification of *AIB1 *gene in different passages of ER positive MCF-7 cell lines remains at high levels of 18–23 fold of control. Loss of estrogen dependence in LCC1 is accompanied by moderate decrease in *AIB1 *gene amplification (13.5 fold in LCC1 versus 22.2 fold in MCF-7). The level of *AIB1 *gene amplification was reduced to 14.6 fold when the estrogen-independent cells became resistant to antiestrogen 4-OH TAM treatment (LCC2). Similar decrease in amplification (15.6 fold of control) of *AIB1 *gene was observed in estrogen-independent cell line that has gained antiestrogen resistance to both non-steroid, 4-OH TAM, and steroid, ICI 182,780 antiestrogens (LCC9) (Table 1).

### Somatic instability of poly Q encoding region of AIB1 gene in breast cancer cell lines

The polymorphic poly Q encoding region of *AIB1 *contains CAG repeat that is frequently interrupted by CAA's. The poly Q region is part of the histone acetyltransferase domain. It is also where the recruitment and interactions with other components of the transcription activator complex takes place. In order to investigate if qualitative alteration in this region accompanied the quantitative change of *AIB1 *gene in breast cancer cell lines, we cloned and sequenced the poly Q encoding region of the gene. The cloning/sequencing technique resolved the heterogeneous poly Q encoding sequences into distinct sequences, thus allowing the analysis at the single molecule level. At least 8 clones from each cell line were selected and sequenced. Theoretically, there should be only 2 distinct sequence patterns if the cell line is heterozygous for *AIB1 *allele and one distinct sequence pattern if it is homozygous. However, 18/25 (72%) (data partially shown in Table 2) of the cell lines contain at least one poly Q encoding sequence pattern that represents less than 20% of the sequenced clones of the cell line. These results suggest that the under-represented sequences probably arise from the parental sequence by somatic mutation. Indeed these rare sequences differ from their parental sequence by one base pair substitution (CAG to CAA) or by insertion or deletion of CAGs. The high degree (72% of the cell lines) of somatic instability is probably characteristic for cancer cell lines since it only occurs in less than 5% (2/43) of the normal controls. We analyzed normal A1N4 cell line at two different times. The first time, ten clones of A1N4 cell line were sequenced. Pattern 2 (Table 2), (CAG)_6_CAA(CAG)_9_(CAACAG)_3_(CAACAGCAG)_2_CAA, was found in 4 clones, and pattern 17, (CAG)_4_CAA(CAG)_9_(CAACAG)_3_(CAACAGCAG)_2_CAA was found in 6 clones. The second time, 31 clones were sequenced. Sixteen had pattern 2 and 15 had pattern 17. There was no occurrence of "extra" poly Q encoding sequence. These results suggest that the occurrence of rare sequences is not due to cloning/PCR artifact.

Association between poly Q length or its specific encoding sequence with *AIB1 *amplification was not recognized (Table 2). Somatic instability occurred in all variants of MCF-7 cell line, although they all maintain the parental allele as the predominant coding sequences (pattern 1). Two new sequences arise by insertion of 2 and 3 CAGs in passage 19. Another two new sequences occur in passage 72 by deletion of 1 and 2 CAG repeats. Similar somatic mutations occur in cell lines LCC1, LCC2, LCC9, LY-2 and R27. These mutations seem to occur randomly and independently in each cell line. There is not any single sequence that occurs more frequently than others, except pattern 5, which occurs 3 times by losing 1 CAG directly from the parental sequence.

AK47 was derived from ZR-75-1 by losing its ER activity. During the establishment of the cell line, additional somatic mutations occurred in the polyglutamine region (patterns 11 and 16). The poly Q encoding sequence of *AIB1 *gene seems to be quite unstable in MDA-MB435 cell line. It has 4 distinct poly Q encoding sequence patterns with pattern 9 as the predominant one. Its variant LCC6 had a total of 7 different sequence patterns. These data are consistent with the genomic instability that is characteristic for cancer cells. Although poly Q encoding sequence patterns do not seem to directly link to *AIB1 *gene amplification, it is possible that the alteration in poly Q length affects protein-protein interaction, thus, the transactivation activity of *AIB1*. While most alterations do not change poly Q length significantly, rare sequence pattern in LCC2 with much shorter (only 14 repeats) poly glutamine tract may affect the co-transactivating activity of *AIB1 *gene.

## Discussion

Genetic and clinical phenotypic heterogeneity is the prominent characteristic of breast cancer. Multiple genetic alterations contribute to breast cancer development and progression [35,36]. The occurrence of DNA amplifications in breast cancer had been studied by Southern blot [1], FISH (fluorescence in situ hybridization) [4] and CGH methods (comparative genomic hybridization) [37-39]. We developed real time quantitative PCR method to more accurately assess the amplification of *AIB1 *gene in breast cancer cell lines. Amplification of *AIB1 *in breast cancer cell lines; BT-474 and MCF-7 were first reported by Guan et al. [3]. By FISH analysis, Anzick et al. [7] observed >20 fold amplification of *AIB1 *gene in three ER positive breast cancer cell lines (BT-474, MCF-7 and ZR-75-1) and, to a lesser extent, in 10% primary breast tumors. In this study we did not detect significant amplification in ZR-75-1 cell line. The discrepancy may be explained by a different source of the cell line or by spontaneous change of the cell line during passages. In addition, FISH analysis is restricted to a few cells, whereas real time qPCR analysis measures the gene in the overall DNA extract. Glaeser et al. [2] used the quantitative differential PCR to determine the amplification level of *AIB1 *and found no amplification in breast or endometrial carcinomas. These methods did not give actual copy numbers of the gene. In this study, we used real-time Q-PCR to determine the level of *AIB1 *amplification. The ability of real-time Q-PCR to detect the fluorescent signal from degraded sequence specific TaqMan probe at the very beginning period of exponential stage offered an accurate way of DNA quantification. When compared to CGH, FISH and Southern blot analysis, this method has the advantage of high sensitivity, reproducibility, and efficiency.

If only the original cell lines were counted, the *AIB1 *gene was amplified in 2 out of 9 ER positive and none of 10 ER negative cell lines. Higher degree (22%) of *AIB1 *amplification in ER positive breast cancer cell lines may suggest the association between *AIB1*gene amplification and ER status. This is further supported by the observation that the *AIB1 *amplification moderately decreased when cells became ER independent as LCC1, LCC2 and LCC9 consistent with the role of AIB1 in ER-dependent signaling. All MCF-7 variant cell lines maintain high level of *AIB1 *gene amplification of the parental cells. Additional gain of resistance to an antiestrogen, ICI182,780, does not have significant effect on *AIB1 *amplification (from LCC1 to LCC9). Similarly, resistance to 4-OH TAM is not consistently accompanied with the change in *AIB1 *gene amplification. LY-2, R27, and LCC2 were all selected from estrogen dependent MCF-7 cells against high dose of 4-OH TAM. *AIB1 *gene amplification in LY-2 and R27 cell lines remained almost unchanged, whereas in LCC2, *AIB1 *gene amplification is moderately decreased. These data suggest that resistance to 4-OH TAM does not necessarily affect *AIB1 *gene amplification. It should be noted that in LCC2 there is a short poly Q containing mutant *AIB1 *and lower expression of the gene may be compensated by the increase in co-transactivation activity of the mutant protein. Our observation of variations in *AIB1 *gene amplification in various derivatives of MCF-7 cell clines is consistent with the previous report in which several MCF-7 sublines were shown to have the capacity to generate clonal heterogeneity. This represents an important selective advantage in MCF-7 in leading to aggressive and metastatic forms of the disease [40].

Besides the quantitative regulation of *AIB1 *gene in breast cancer cell lines, the *AIB1 *gene contains CAG repeat region which is a target for genetic instability in tumor progression. Large expansion of triplet repeat occurs in various neurodegenerative diseases [41-43]. These abnormal proteins form large aggregates that have been shown to tie up transcription factors that bind poly Q such as CREB [44]. The poly Q tract in the androgen receptor (AR) gene is unique in that the large expansion of poly Q encoding CAG repeat causes the X-linked spinal bulbar muscular atrophy (SBMA, or Kennedy Disease) [19,20], but short poly Q of AR is correlated with hormone-dependent transactivation [19,20] and more aggressive form of cancer [21,45]. *AIB1 *shares several structural/functional similarities with AR. Both genes are involved in nuclear receptor mediated regulation of gene expression. Due to frequent interruption with CAA's, large expansion of the triplet was not observed in *AIB1 *gene. This region, however, was quite unstable as evidenced by frequent CAA/CAG changes and small insertions and deletions. The PCR/cloning strategy allows us to investigate the polymorphic poly Q encoding region at single molecule level. Since we only sequenced a small number of clones from each individual, we cannot exclude the possibility that the under-represented alleles may be lost in PCR/cloning/sequencing process. Several distinct poly Q encoding sequence patterns were observed in LCC-6, T47D, and MDA-MB157. T47D is a cell line of notable genetic instability that was observed in the estrogen receptor gene [46]. LCC6 cell line which formed ascites was a more aggressive form of MDA-MB435 [30]. Since various rare poly Q encoding sequences seem to arise randomly and independently in different cell lines regardless of the *AIB1 *gene amplification levels, we attribute these somatic mutations to genetic mutability in cancer cells.

## Conclusion

The poly Q encoding sequence of *AIB1 *gene is genetically unstable and is an easy target for somatic mutations in cancer cells.*AIB1 *gene amplification occurs in only a small fraction of ER positive primary breast tumors and breast cancer cell lines. AIB1 gene amplification has not been found in ER negative primary tumor or breast cancer cell lines. Gain of estrogen independence and resistance to steroid antiestrogen may be accompanied by moderate decrease of *AIB1 *gene amplification.

## Abbreviations

4-OH TAM = 4-hydroxy tamoxifen; ACTR = Activator of Retinoid and Thyroid Receptors; *AIB1 *= *Amplified in Breast Cancer *gene *1*; bHLH-PAS = basic helix-loop-helix -Per-ARNT-Sim; *β*_2_*-M *= *β*_2_*-microglobulin *gene; CBP = CREB binding protein; CREB = cAMP-responsive element binding protein; ER = estrogen receptor; FAM = 6-Carboxy-Fluorescein; RAC3 = Receptor-Associated Co-activator 3; TRAM-1 = Thyroid Hormone Receptor Activator Molecule1; TAMRA = 6-Carboxy-Tetramethyl rhodamine

## Competing interests

The author(s) declare that they have no competing interests.

## Authors' contributions

LW participated in the conception and design of study, acquisition, analysis, and interpretation of results, and the final write up of the manuscript. PD carried out the cloning/sequencing experiment. JL extracted the DNA. ML collected tumors and specimens. RC provided various cell lines and participated in discussion. VN performed the real time quantitative PCR analysis. All authors read and approved the final manuscript.

## Pre-publication history

The pre-publication history for this paper can be accessed here:



## Figures and Tables

**Table 1:** The ER status, *AIB1 *amplification, and poly Q encoding sequences in breast cell lines

Cell line ^*a*^	ER status	*AIB1 *copy number ratio (tumor/normal)	Number of sequence patterns
MCF-10A	-	1.1	3
MCF-10A neo	-	1.3	3
**A1N4**^*b*^	-	2.0	2
**AK-47**	-	1.4	3
**MDA-MB435**	-	1.3	4
**LCC6**	-	2.0	7
MDA-MB157	-	1.1	6
MDA-MB134V	-	1.4	2
MDA-MB231N	-	1.2	2
HBL100	-	1.2	1
ZR-75-30	-	1.7	1
HS 578T	-	1.9	2
HS 578BST	-	1.1	2
**MCF7**	+	22.2	1
**MCF-7 P19**	+	18.7	3
**MCF-7 P72**	+	22.8	3
**LY-2**	+	19.9	2
**R27**	+	19.4	2
**LCC1**	+	13.5	3
**LCC2**	+	14.6	2
**LCC9**	+	15.6	2
BT474	+	4.9	2
BT-483	+	1.9	4
BT20	+	1.7	2
MDA-MB468	+	1.7	2
**ZR-75-1**	+	1.5	2
MDA-MB361	+	1.3	2
T47D	+	1.0	5
MDA-MB330	+	0.9	2

^*a*^LCC1: estrogen independent and responsive which is selected for growth in vivo without estrogens; LCC2: selected from LCC1 by treatment with 4-OH TAM; LCC9: selected from LCC1 by treatment with ICI 182780, resistant to ICI182780 and 4-OH TAM:, LY-2: selected for resistance to 4-OH TAM, KEO and LY 117018; R27: able to grow in the presence of 4-OH TAM. LCC6 is the more aggressive variant of MD-MB435. AK-47 is the variant derived from ZR-75-1. A1N4 is a normal breast cell line.

^*b*^Cell lines listed in Table 2 are in bold.

**Table 2:** Poly Q sequence patterns and *AIB1 *amplification level in MCF7 and its variants

Cell line^*a*^	amplification	Sequence patterns	Pattern	(Q)n	Frequency
MCF-7	22.3	**(CAG)_3_CAA(CAG)_9_(CAACAG)_3_(CAACAGCAG)_2_CAA**	1	26	7/7
MCF-7 P19	18.7	**(CAG)_3_CAA(CAG)_9_(CAACAG)_3_(CAACAGCAG)_2_CAA**	1	26	7/9
		(CAG)_6_CAA(CAG)_9_(CAACAG)_3_(CAACAGCAG)_2_CAA	2	29	1/9
		(CAG)_5_CAA(CAG)_9_(CAACAG)_3_(CAACAGCAG)_2_CAA	3	28	1/9
MCF-7 P72	22.8	**(CAG)_3_CAA(CAG)_9_(CAACAG)_3_(CAACAGCAG)_2_CAA**	1	26	5/7
		(CAG)_3_CAA(CAG)_8_(CAACAG)_3_(CAACAGCAG)_2_CAA	4	25	1/7
		(CAG)_3_CAA(CAG)_7_(CAACAG)_3_(CAACAGCAG)_2_CAA	5	24	1/7
LCC1	13.5	**(CAG)_3_CAA(CAG)_9_(CAACAG)_3_(CAACAGCAG)_2_CAA**	1	26	7/9
		(CAG)_3_CAA(CAG)_8_(CAACAG)_3_(CAACAGCAG)_2_CAA	4	25	1/9
		(CAG)_3_CAA(CAG)_14_(CAACAG)_2_(CAACAGCAG)_2_CAA	6	29	1/9
LCC2	14.6	**(CAG)_3_CAA(CAG)_9_(CAACAG)_3_(CAACAGCAG)_2_CAA**	1	26	4/5
		CAG(CAACAG)_3_(CAACAGCAG)_2_CAA	7	14	1/5
LCC9	15.6	**(CAG)_3_CAA(CAG)_9_(CAACAG)_3_(CAACAGCAG)_2_CAA**	1	26	7/8
		(CAG)_3_CAA(CAG)_2_CAA(CAG)_9_(CAACAG)_3_(CAACAGCAG)_2_CAA	8	29	1/8
LY-2	19.9	**(CAG)_3_CAA(CAG)_9_(CAACAG)_3_(CAACAGCAG)_2_CAA**	1	26	9/10
		(CAG)_6_CAA(CAG)_8_(CAACAG)_3_(CAACAGCAG)_2_CAA	9	28	1/10
R27	19.4	**(CAG)_3_CAA(CAG)_9_(CAACAG)_3_(CAACAGCAG)_2_CAA**	1	26	8/9
		(CAG)_3_CAA(CAG)_8_(CAACAG)_3_(CAACAGCAG)_2_CAA	4	25	1/9
MDA-MB435	1.3	(CAG)_3_CAA(CAG)_2_CAA(CAG)_11_(CAACAG)_2_(CAACAGCAG)_2_CAA	10	29	2/8
		(CAG)_6_CAA(CAG)_8_(CAACAG)_3_(CAACAGCAG)_2_CAA	9	28	3/8
		(CAG)_3_CAA(CAG)_9_(CAACAG)_3_(CAACAGCAG)_2_CAA	1	26	2/8
		(CAG)_6_CAA(CAG)_8_(CAACAG)_2_(CAACAGCAG)_2_CAA	11	26	1/8
LCC6	2.0	(CAG)_3_CAA(CAG)_2_CAA(CAG)_10_(CAACAG)_2_(CAACAGCAG)_2_CAA	12	28	1/9
		(CAG)_3_CAA(CAG)_2_CAA(CAG)_6_(CAACAG)_3_(CAACAGCAG)_2_CAA	13	26	1/9
		(CAG)_6_CAA(CAG)_8_(CAACAG)_3_(CAACAGCAG)_2_CAA	9	28	3/9
		(CAG)_3_CAA(CAG)_2_CAA(CAG)_10_(CAACAG)_3_(CAACAGCAG)_2_CAA	14	30	1/9
		(CAG)_6_CAA(CAG)_8_(CAACAG)_3_(CAACAGCAG)_2_CAA	15	28	1/9
		(CAG)_3_CAA(CAG)_2_CAA(CAG)_11_(CAACAG)_2_(CAACAGCAG)_2_CAA	10	29	1/9
		(CAG)_3_CAA(CAG)_2_CAACAGCAA(CAG)_8_(CAACAG)_2_(CAACAGCAG)_2_CAA	16	28	1/9
ZR-75-1	1.5	(CAG)_6_CAA(CAG)_9_(CAACAG)_3_(CAACAGCAG)_2_CAA	2	29	6/7
		(CAG)_5_CAA(CAG)_9_(CAACAG)_3_(CAACAGCAG)_2_CAA	3	28	1/7
AK-47	1.4	(CAG)_6_CAA(CAG)_9_(CAACAG)_3_(CAACAGCAG)_2_CAA	2	29	2/8
		(CAG)_6_CAA(CAG)_8_(CAACAG)_3_(CAACAGCAG)_2_CAA	11	28	5/8
		(CAG)_5_CAA(CAG)_8_(CAACAG)_3_(CAACAGCAG)_2_CAA	15	27	1/8
A1N4	2.0	(CAG)_6_CAA(CAG)_9_(CAACAG)_3_(CAACAGCAG)_2_CAA	2	29	20/41
		(CAG)_4_CAA(CAG)_9_(CAACAG)_3_(CAACAGCAG)_2_CAA	17	27	21/41

^*a*^LCC1: estrogen independent and responsive which is selected for growth in vivo without estrogens; LCC2: selected from LCC1 by treatment with 4-OH TAM; LCC9: selected from LCC1 by treatment with ICI 182780, resistant to ICI182780 and 4-OH TAM:, LY-2: selected for resistance to 4-OH TAM, KEO and LY 117018; R27: able to grow in the presence of 4-OH TAM. LCC6 is the more aggressive variant of MD-MB435. AK-47 is the variant derived from ZR-75-1. A1N4 is a normal breast cell line. The parental sequence patterns are in bold.
